# Validation of a Web-Based, Time-Use Application to Assess Children’s School Meal Intakes: My E-Diary for Activities and Lifestyle (MEDAL)

**DOI:** 10.3390/nu13113790

**Published:** 2021-10-26

**Authors:** Sarah Yi Xuan Tan, Airu Chia, Ray Sugianto, Huiying Eunice Tong, Ian Yi Han Ang, Lynette Pei-Chi Shek, Seang Mei Saw, Falk Müller-Riemenschneider, Mary Foong-Fong Chong

**Affiliations:** 1 Saw Swee Hock School of Public Health, National University of Singapore and National University Health System, Singapore 117549, Singapore; sarahtan@u.nus.edu (S.Y.X.T.); airu-chia@nus.edu.sg (A.C.); ray.sugianto@u.nus.edu (R.S.); the@nus.edu.sg (H.E.T.); yha2103@columbia.edu (I.Y.H.A.); saw.seang.mei@seri.com.sg (S.M.S.); falk.m-r@nus.edu.sg (F.M.-R.); 2 Department of Paediatrics, Yong Loo Lin School of Medicine, National University of Singapore, Singapore 119228, Singapore; lynette_shek@nuhs.edu.sg; 3 Khoo Teck Puat-National University Children’s Medical Institute, National University Health System, Singapore 119074, Singapore; 4 Singapore Eye Research Institute, Singapore National Eye Centre, Singapore 168751, Singapore; 5 Duke-NUS Medical School, Singapore 169857, Singapore; 6 Singapore Institute for Clinical Sciences, Agency for Science, Technology and Research, Singapore 117609, Singapore

**Keywords:** children, dietary intake, web-based application, self-report, validity, meal photography

## Abstract

My E-Diary for Activities and Lifestyle (MEDAL), a web-based application, was developed to assess the diets of children. This study examined the validity of school recess meals reported by children on MEDAL, using meal photography as the reference. Recess meals were photographed by trained researchers, and food items and portion sizes of recess meals reported on MEDAL were compared to recess meal photos. Validity was assessed by percentages of match, omission and intrusion for food items and percentages of the match, underestimation and overestimation for portion sizes. The Mann–Whitney test and the Wilcoxon matched-pairs signed-rank test examined if sex, school and day of recording influenced the validity of food item reporting. We found that participants (*n* = 33, aged 10–11 years) recalled 60.2% of food items consumed at recess accurately (matches); omissions (24.6%) were more common than intrusions (15.2%). Omissions tended to be side dishes, and intrusions tended to be high-calorie items. Sex, school and day of recording did not influence validity. For food portion sizes, 58.3% of items were accurately reported. Overestimations (33.3%) were more common than underestimations (8.3%). In conclusion, these children were able to report food items consumed during school recess meals using MEDAL, albeit with limitations on the degree of accuracy.

## 1. Introduction

Understanding children’s dietary choices will aid in the development of targeted strategies aimed at fostering healthy dietary habits as they transition into adulthood [1]. However, assessing schoolchildren’s food intake can be challenging. Parents often lack first-hand knowledge of their child’s out-of-home food intake, thus limiting the accuracy of proxy-food reporting [2]. On the other hand, traditional pen-and-paper dietary assessment methods such as 24-h recall, food diaries and food frequency questionnaires involve lengthy and extensive interviews or completion of questionnaires, which can be burdensome for children [3] and affect their motivation to complete the assessment, resulting in low compliance.

Information technology presents a promising opportunity to circumvent the challenges of collecting self-reported dietary intake information from children. Interactive and animated features can facilitate engagement and influence the child’s interest and motivation to complete the assessment [3]. The ability to present food and portion size images on the same screen may also improve the accuracy of portion size reporting among children and adolescents [4]. Additionally, the use of web-based dietary assessments streamlines data collection and evaluation processes, thus improving cost- and time-effectiveness [5].

Several countries have leveraged information technology to develop multimedia (e.g., a combination of text, audio, still images, animation, video and interactivity elements) and web-based assessments (i.e., administered online) to collect self-reported dietary information from children [6,7,8,9,10,11], but these are limited in Asia. In Singapore, research in recent years has largely only published dietary data on younger children aged five years and below [12], adolescents at least 13 years of age [13] and adults [14], with limited information on the food intakes of older children between 5 to 13-years-old [15]. To bridge this gap, a web-based diet and activity diary, named My E-Diary for Activities and Lifestyle (MEDAL), was developed to potentiate the collection of time-use information from children at least 10 years of age. It prompts users to recall and report dietary intakes and activities in a 24-h day and collects data over multiple days [16]. Usability tests have demonstrated that MEDAL is acceptable for use among 10 to 11-year-old children in Singapore [16].

However, the difficulty in predicting the accuracy of children’s self-reports was highlighted in the validation of other web-based dietary assessments, where varying degrees of reporting errors for both food item and portion size reporting were demonstrated [6,7,9,10,11]. A multitude of factors can influence the accuracy of children’s self-reports, such as reliance on memory [17], social desirability [18], cognitive ability [19], knowledge of foods and preparation methods, and if the foods served to the child were presented the same way as the food portion pictures provided during the diet assessment [4,20,21]. Hence, there is a need to evaluate the accuracy of self-reported food intake collected using MEDAL.

Comparing the children’s self-reports to other measures of food intake capture (criterion measure) such as direct observation or meal photography, and presenting results by number and type of reporting errors (i.e., omissions and intrusions of food items), is a common method for validating dietary assessments for children [22]. In this study, we used meal photography as the criterion measure to assess the accuracy of self-reported dietary intake of the children’s meals in school. This method was used to validate dietary assessment such as the Fruit and Vegetable Recall Questionnaire [23] and Web-based Dietary Assessment Software for Children (WebDASC) [24] among children in western populations. It is free from recall bias and can be conducted by a single photographer, making it advantageous as a cost- and labour-efficient method of objectively assessing dietary intake [25,26]. The school environment provides an ideal setting to conduct meal photography for validating the children’s self-reported intake [27] as all children consume their recess meal in the same location (i.e., the school canteen), thus meal photography can be conducted with logistical ease. Furthermore, food intake in school occurs relatively unsupervised. Food consumed in school would have to be reported by the children with limited assistance from their parent, caregiver or teachers, thus allowing a more accurate assessment of their ability to independently report their dietary intake.

We hypothesise that children can report their recess meals on MEDAL, albeit with some limitations in accuracy. This study evaluated the validity of food items consumed during school recess meals reported by 10 to 11 years old children on MEDAL, in reference to recess meal photos captured by a researcher.

## 2. Materials and Methods

### 2.1. Participants

Students aged 10 to 11 years from the Primary Five level were recruited from two co-educational, government schools in Singapore (hereafter referred to as Schools A and B) between April–September 2019. Students were eligible if they were able to understand and respond to the assessment in English.

There were seven and six Primary Five classes from School A and School B respectively. For logistical reasons, two classes from each school were randomly selected to participate in this diet validation study, while students of the remaining classes were involved in validating movement behaviours (e.g., use of accelerometer to validate self-reported movement behaviours).

The Singapore Ministry of Education (MOE) approved data collection from the two primary schools in Singapore, and the National University of Singapore Institutional Review Board approved the study (reference code: S-18-088). Informed written consent was obtained from parents or guardians of all student participants. All student participants provided verbal assent, which was observed and formally recorded.

### 2.2. Study Procedures

#### 2.2.1. Demonstration Session

A standard protocol was developed to ensure consistency in how the validation studies were conducted across the two schools.

All students attended a demonstration session conducted in their school’s computer laboratories. Trained researchers demonstrated how to log in and navigate MEDAL. The students were instructed to record their diet and daily activities over two specified weekdays and two weekend days independently. Participants in the present diet validation study mostly completed MEDAL entries at home, but some did not have access to the internet at home and completed their entries in school, using school computers the next school day.

Participants in this diet validation study were instructed to report to a meal photography booth set up at the back of their school canteen during their recess breaks on the two specified weekdays they were tasked to complete MEDAL. This included all participants who bought meals, snacks, drinks or single items (e.g., buns) from the school canteen, brought food and/or drinks from home, or those who were not eating. This enabled the subsequent reporting of recess meals in MEDAL by the children to be assessed in relation to food intake determined objectively by meal photography.

#### 2.2.2. Recess Meal Photography

**Figure 1:**
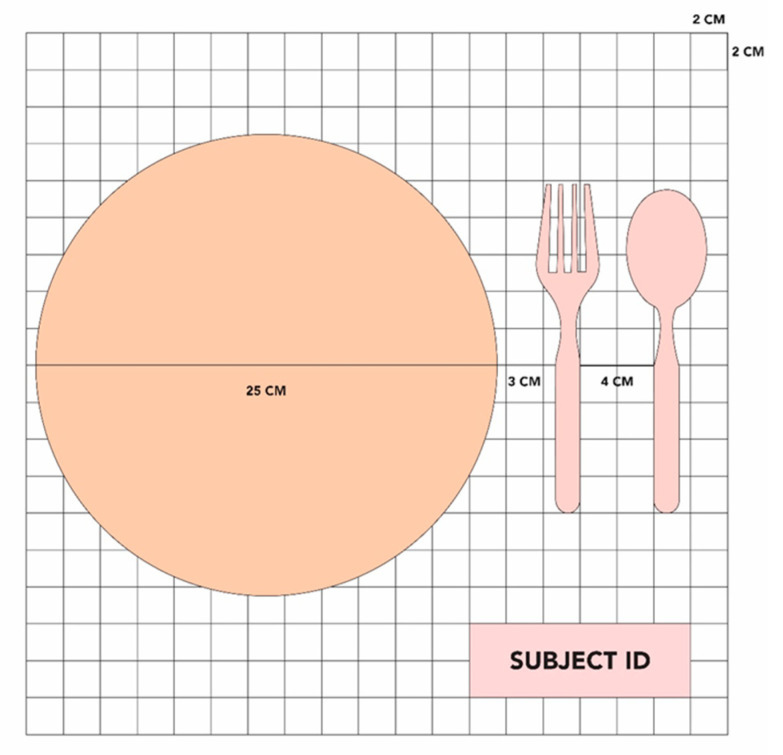
School meal photography booth setup.

In Singapore, all primary school children are given a midmorning recess break at a set time, where foods that are store-bought or brought from home are consumed in one location: the school canteen. Recess meal photography was used as the objective reference method for this study. Recess periods for Schools A and B were 10.40–11.10 AM and 10.15–10.45 AM respectively (30 min). Photos of all school-bought or brought-from-home recess meals were taken by trained researchers using research smartphones mounted on a tabletop tripod. The camera lens of each smartphone was positioned at approximately 26 cm from the table’s surface, with the smartphone angled at 45° relative to the horizontal tabletop [28,29]. A reference plate was placed beside the reference cutleries (i.e., spoon and fork), atop a 2 cm × 2 cm gridded, ruled mat in front of the tripod. This setup is illustrated in Figure 1.

Participants were asked to approach the meal photography booth before consuming their meal items. At the booth, the research staff noted each participant’s unique subject identification code and details of their recess meal (e.g., fillings of buns that were difficult to photograph). Each participant’s meal was placed on top of the reference plate with their unique study ID written and positioned within the camera frame in order to capture the before-meal photo. Where necessary, recess meal items were rearranged to ensure that all the meal components were conspicuous when photographed. Participants were reminded to return to the booth after they have completed their meals. After-meal photo taking was then conducted using a similar procedure to before-meal photo taking. Researchers also asked if the participants spilled, gave away or received food, or saved food for subsequent consumption before capturing the after-meal photos. If the participant had a second serving, the entire meal photo-taking procedure was repeated. Additionally, two researchers walked around the canteen to remind students involved in the diet validation study to report to the meal photography booth when they have finished eating.

#### 2.2.3. The MEDAL Application for Diet Assessment

The conceptualisation, design, and usability of MEDAL were described elsewhere [16]. In brief, MEDAL is a self-administered, web-based application that captures information on multiple lifestyle behaviours of children through the completion of a four-day, time-use diet and activity diary. Participants can choose from six broad activities: “Shower/Wash Up”, “Travelling”, “Eat & Drink”, “Nap/Sleep”, “Sitting Activities” or “Active Activities”.

**Figure 2:**
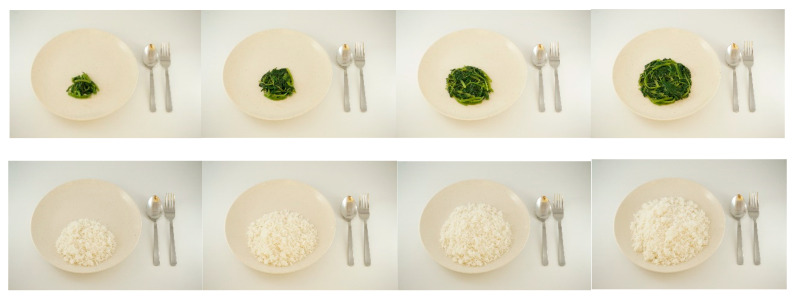
Images for dark green leafy vegetables (**top**), and white rice (**bottom**), presented for portion size selection in MEDAL.

When participants chose “Eat & Drink”, they were prompted to select the food they consumed from 88 food and drink icons available [16]. This list of food and drink items was compiled from food/drink items frequently consumed by children and mothers from the GUSTO mother–offspring birth cohort in Singapore, as well as based on consultations with experts in dietary assessment [16]. Participants were also allowed to manually enter foods that are not listed in the application [16]. For each chosen food or drink item, participants were provided with four pictorial portion size options to select the one that best correspond to the amount they consumed (Figure 2) [16]. For manually entered food items, participants reported if they consumed “half a standard portion”, “one standard portion”, “one-and-a-half standard portion”, or “two standard portions” [16]. The portion size descriptions were kept broad as these manually entered food items could come from a variety of foods.

#### 2.2.4. Demographic Data Collection

Apart from age and sex information that was collected through MEDAL, participants’ anthropometric measurements were measured by the participating schools and data was shared with the researchers. Body mass index (BMI) of the participants were then calculated using the formula: weight (kg)/(height (m) × height (m)) and the children categorised into underweight (<5th percentile), within healthy weight range (5th≤90th percentile) and overweight (≥90th percentile) categories based on the age- and sex-specific BMI reference data for Singaporean children [30]. Information on participants’ internet access at home was collected from the consent form.

### 2.3. Data Processing

The accuracy of the participants’ self-reported recess meals was determined by comparing self-reports on MEDAL to the meal photos taken by researchers (criterion measure). Only weekday data were used in the analysis for the current paper as recess meal photography was only conducted on weekdays. Each participant’s before- and after-meal photos were matched to their recess meals reported in MEDAL using their unique subject identification code to create “sets”. Items reported on MEDAL were considered recess meals if participants identified “school” as the location where these items were consumed, and the date and time of the reported recess meal coincided with that of the meal photography session. Sets, where either the MEDAL recess meal entry or recess meal photos were not available, were considered “missing” and excluded case-wise for the validation analysis as matching of MEDAL entries to recess meal photos would not be possible.

The food items on MEDAL consisted of single items (e.g., drinks, snacks, or desserts), composite meals, or components of mixed meals. A composite meal would comprise a staple food item with ingredients from multiple food groups (e.g., fried rice comprising rice, eggs, meat, peas, and carrots). A mixed meal would comprise a staple food item (e.g., white rice) served with a variable number of side dishes (e.g., stir-fried broccoli, steamed chicken, etc.). All food items were categorised into 11 food groups (i.e., Bread, spreads, and cereals; Curry; Drinks; Eggs and tofu; Fruits; Meat and fish; Milk, cheese, and yogurt; Noodles, pasta, and potatoes; Rice and porridge; Snacks and desserts; and Vegetables and mushroom) for analyses at the food group level.

Food item reporting: A trained researcher compared each of the food items reported in MEDAL to the food items identified from the before and after meal photos and classified it as a match, omission, or intrusion based on previously established definitions [11]. A food item match occurred when the item reported in MEDAL was present in the meal photo. This was further categorised into a strict match (when a food item reported in MEDAL exactly matched the item in the meal photo) or a loose match (when a food item reported in MEDAL was not identical to the item in the meal photo but belonged to the same food group) [10,22]. An omission occurred when a food item was not reported in MEDAL but was present in the meal photo. An intrusion occurred when a food item was reported in MEDAL but was not present in the meal photo.

Match, omission and intrusion rates were calculated using the following formula [11]:

Match rate = (total number of items classified as “strictly-matched” and “loosely-matched”)/(sum of matches, omissions and intrusions) × 100%.

Omission rate = (number of items classified as an “omission”)/(sum of matches, omissions and intrusions) × 100%.

Intrusion rate = (number of items classified as an “intrusion”)/(sum of matches, omissions and intrusions) × 100%.

Portion size reporting: Additionally, the trained researcher evaluated the accuracy of portion sizes reported by the participants by performing a portion size analysis [23]. Only food items reported on MEDAL that were considered a match were included for portion size analysis. Manually entered food items were included in portion size analysis if they were considered a match and if their reported portion sizes were clearly defined. From the meal photos, the portion of each food item consumed during recess was estimated by the researcher using the gridded, ruled mat, reference plate and cutleries, and information on whether participants spilled, gave away, or received food. The estimated portion size was coded with reference to the four portion size options for each food item provided on MEDAL. The coded portion size for each food item was compared to the portion size option selected by the participant. A portion size match occurred when the portion size reported on MEDAL by the participant was the same as that coded by the trained researcher using the meal photos. Underestimation occurred when the portion size reported was less than the portion size coded by the researcher. Overestimation occurred when the portion size reported exceeded the portion size coded by the researcher.

In a pilot study, inter-rater agreement between two researchers (determined using Cohen’s kappa statistics) demonstrated near-perfect and substantial agreement for food item identification (*n* = 92, k = 0.85) and portion size estimation (*n* = 81, k = 0.70) respectively (Table A1 in Appendix A). Thus, one researcher conducted the data processing and analysis in the present validation study.

### 2.4. Statistical Analyses

As the data were found to be non-parametric, tests for differences between included and excluded participants, and comparison of baseline characteristics between included participants of Schools A and B were performed using Pearson χ^2^ tests. Criterion validity was examined and presented as percentages of food item match, omission, and intrusion, as well as portion size match, underestimation, and overestimation. Mann–Whitney test was applied to determine if participants of different sex and school differed in reporting accuracy. To compare if recess meals reported on MEDAL on the two recess meal photography days differed in reporting accuracy, Wilcoxon matched-pairs signed-ranks test was applied to compare the accuracy of reports made on the first and second day of recess meal photography for participants who have complete sets of data for both days. Sensitivity analyses were also conducted using the subset of participants with access to the internet at home. Statistical analyses were performed using Stata SE (version 14.2, 2016, StataCorp, College Station, TX, USA). All tests were two-sided, and *p*-values < 0.05 were considered statistically significant.

## 3. Results

**Figure 3:**
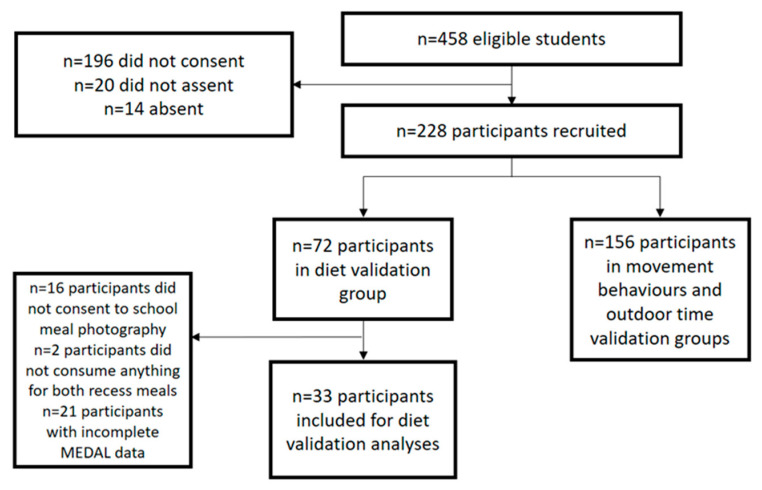
MEDAL diet validation participant flow diagram.

Of 228 students recruited from the two participating schools, 72 participants were randomly selected to be in the present diet validation study, where 56 participants provided written consent for their recess meals to be photographed. Among these, 33 participants (median 11.0 years, interquartile range 10.0–11.0 years) who consumed at least one food item during recess and reported their recess meal in their MEDAL entry on at least one validation day were included for analyses (59% response rate). Participants who did not consume any food or drink items during recess on both days (*n* = 2) and participants who did not have any MEDAL entry that corresponded to these recess periods (*n* = 21) were excluded. The characteristics (age, school, sex, BMI-for-age and internet access) of included and excluded participants did not differ significantly (*p* ≥ 0.05) (Table A2). Figure 3 presents the participant flow diagram.

Participants (*n* = 33) from Schools A and B did not differ significantly in age, sex, BMI-for-age, accessibility to the internet, and the number of days of completed recording on MEDAL (*p* > 0.05). Slightly more than half of the final sample was female (54.5%), and a large proportion of the final sample was of healthy weight (78.8%), had access to the internet at home (87.9%), and completed one out of two days of recording (63.6%) (Table 1).

### 3.1. Food Item Reporting Accuracy

Table 2 describes the match, omission, and intrusion rates of the recess meal items consumed. Of 118 food items included for the analysis, the total match rate (combination of strict and loose matches, 49.2% and 11.0% respectively) was 60.2%, omission rate was 24.6%, and intrusion rate was 15.2%. At the food group level, carbohydrate-based items from the “Breads, spreads, and cereals”, “Noodles, pasta, and potatoes” and “Rice and porridge” categories were most accurately reported (match rates 76.9–81.8%). About half of “Curry”, “Vegetables and mushrooms” and “Eggs and tofu” items, which are typically side dishes of mixed meals, as well as “Fruits”, were omitted (omission rates 50.0–57.1%). At least one-third of items from the “Drinks” and “Snacks and desserts” categories were intruded (intrusion rates 33.3–42.9%).

Food item match, omission, and intrusion rates were further stratified by sex, school, and day of recording and compared. Reporting accuracy on MEDAL did not significantly differ between sex, school and day of recording (*p* > 0.05). Analysis of the subset of participants with access to the internet at home did not significantly influence reporting accuracy of food item match. These results were presented in Table A3.

### 3.2. Portion Size Reporting Accuracy

Table 3 describes the portion match, underestimation, and overestimation of the recess meal items consumed. Of *n* = 71 food item matches, *n* = 48 food items were eligible for portion size analysis as the remaining had poorly defined portion sizes (e.g., some manually entered food items). Amongst these items, 58.3% had portions that were exactly matched, while 8.3% and 33.3% were underestimated and overestimated, respectively (Table 3). Portion size of food items from the “Meat and fish”, “Drinks” and “Eggs and tofu” categories were most accurately reported (77.8–100% matched). Conversely, half of the food items from the “Fruits” and “Snacks and desserts” categories were more frequently underestimated, and 80–100% of food items from the “Rice and porridge”, “Noodles, pasta, and potatoes” and “Curry” categories were overestimated. The majority of the portion size estimation errors were due to the selection of the adjacent portion size (65.0%, data not shown) and were thus, close to the portions in meal photos. Analysis of the subset of participants with access to the internet at home did not significantly influence reporting accuracy of portion size match (data not shown).

## 4. Discussion

This validation study confirmed our hypothesis; we demonstrated that children in Singapore aged 10 to 11-years-old are able to report food items consumed during their school recess using a web-based application (MEDAL), although with varying degrees of reporting accuracy. The relatively higher rates of strict matches compared to loose matches demonstrated that a higher proportion of food items were accurately identified and reported compared to a smaller proportion of food items that were less accurately identified but at least belonged to the correct food group. Side dishes tended to be omitted, while drinks, snacks and desserts tended to be intruded.

This study is, to our knowledge, the first in Singapore to validate the self-reported dietary information from children of this age group. Our findings were comparable to that of existing studies in children of western populations above the age of 10-years-old who have completed web-based diet recalls of their meals in school without assistance. These studies (mostly from the United States and Brazil) have demonstrated match rates ranging from 37–53% [10,11,22], whereas in our study, match rates were 60.2%. The slightly higher match rates in the present study may be attributed to MEDAL being a time-use diary [16]. Unlike existing web-based diet recalls that fashion 24-h diet recalls [10,11,22], participants were asked to recount their meals and activities in a 24-h day sequentially, from wake to bedtime. This method, known as behaviour chaining, was demonstrated to improve children’s recall [7], and in the present study, may have contributed to the higher match rates compared to the other studies.

Expectedly, as with any diet recall, the children’s self-reported food intakes were not error-free. In the present study, side dishes were most commonly omitted in the self-reports. Errors in self-reported food intake were largely attributed to children’s cognitive ability [31]. They are not only required to pay attention to what they consumed but are expected to understand what information is being asked of them and retain and retrieve this information when formulating their response to the dietary assessment [19]. Certain foods that are less commonly eaten or that were side dishes or additions to their meals (e.g., condiments) might thus be less salient during recalls and are expectedly more prone to being omitted in self-reports. These factors have previously been identified to account for misreporting of dietary information among children in western populations [2,19,32], and even adults [33].

Another factor leading to misreporting is the information retention period. Among children aged 8–11-years-old, retention period was associated with dietary reporting accuracy; reporting accuracy was higher when children recalled their breakfast and lunch meals in school directly after lunch (short retention period), compared to after breakfast the next morning (long retention period) [2,34]. In the present study, the participants were reminded to report their diet and activities at the end of each day. However, as the recess meal occurred in the morning, they may have forgotten details of their recess meal by the end of the day. Compared to studies where retention periods were either shorter (recalled immediately after meal, omission rate 28%) [22] or slightly longer (recalled meal the next day, omission rate 28–36%) [10,11] than the present study, omission rates in the present study were slightly lower (24.6%). For participants without access to computers or the internet at home, completing their entries the next school day using the school computers may have increased the retention interval, thus affecting their ability to report accurately [2,34]. However, our sensitivity analyses suggest that accessibility to the internet at home did not significantly influence reporting accuracy in the present study.

The intrusion rate when children used MEDAL (15.3%) was also either comparable to or lower than that in other studies (intrusion rate 12–30%) [10,11,22]. Food items such as “Drinks” and “Snacks and desserts” items were intruded more often than food items from the other categories. Although it has been acknowledged that sources of intrusions are understudied and unclear [35], the literature suggests that intruded items might have been confused with memories of items served to or encountered by the participant before or during the study period, mistaking them for being eaten at recess [35,36]. Another possible explanation for the intrusions may be social desirability, where children may report consuming certain foods to be viewed favourably by the researchers and/or their peers [37]. However, as the children in the present study were encouraged to complete their self-reporting in private, the possibility of social desirability bias is less likely [31]. Reduction of omission and intrusion errors in MEDAL may be achieved with proper training and provision of instructions to pay attention to and report details of their meal to improve the quality of self-reported dietary data collected.

Although previous studies in western populations have demonstrated gender differences in reporting accuracy among children of similar age to those in the present study [27,38,39], we did not find any significant difference in reporting accuracy between girls and boys. Girls were expected to be more prone to under-reporting as they were commonly thought to be more susceptible to social desirability and weight pressure [20,40], but this was not observed in the present study sample. An exploration of whether participants of different schools differed in reporting accuracy yielded differences that were non-significant, suggesting that the study site did not confound reporting accuracy. Finally, by comparing the accuracy of the two reported days for participants that have valid sets on both days, although differences were statistically insignificant, reports on the second day demonstrated slightly higher food item match rates and lower rates of errors (i.e., omissions and intrusions) compared to the first day. A potential explanation for this increase in accuracy is that the children gained familiarity in reporting their dietary intakes, therefore omitting fewer food items and achieving higher reporting accuracy as they progressed through the study period.

Besides being able to report what they ate, results from portion size analysis suggest that children were able to provide some estimates of the portion sizes of the food items they consumed, although with varying degrees of accuracy. Among errors, overestimation was more common than underestimation. Although previous studies differ in study design and results are therefore not directly comparable, they generally suggest that portion size reporting by children around the age of 10 years tend to be erroneous and differ by food group or item [9,10,22,24,41]. In the present study, our findings concurred and we found that portion size reporting accuracy also differed by food groups. Interestingly, side dishes that were frequently omitted tend to be estimated accurately when reported. Conversely, while the children could identify and recall the carbohydrate-based items they consumed, the portion size of these items was the least accurate in their estimations.

Various factors may limit the accuracy of portion size estimation, such as when foods do not have a defined surface or did not appear similar to what was depicted in portion pictures [4,21]. For example, carbohydrate-based items (e.g., rice) tend to be topped with side dishes, making the portion of these items less conspicuous and difficult to estimate. Items served as a gravy over food can also be difficult to estimate by children [41], especially also since they are not presented the same way in the food portion pictures provided (i.e., on their own on/in an empty plate or bowl). These factors may implicate the children’s ability to employ abstract reasoning, to translate memories of these items into volumetric measurements, and match that against the portion size photographs to report the amount consumed [31], resulting in the misestimating of these items. However, it was encouraging that majority of portion size estimation errors in the present study were due to the selection of adjacent portion sizes (e.g., portion size 1 instead of portion size 2) (65.0%).

Findings from our study concur with the literature, demonstrating that children around the age of 10 years are able to self-report what they have consumed using web-based applications, albeit with some limitation in accuracy when compared to objective assessments of dietary intake. Additionally, we revealed that certain types of foods such as side dishes of mixed meals and carbohydrate-based items tended to be misreported. This is of particular importance when investigating dietary intakes in the Asian context, as foods consumed are typically mixed meals comprising a carbohydrate item (e.g., rice or noodles) and a variety of side dishes (e.g., meat, vegetables, egg, and/or tofu items). Omission of side dishes and misestimating of portion size of carbohydrate-based items in self-reports could therefore contribute to underreporting of energy intakes and vegetables, one of the key food groups, thereby undermining research on diet and health. This study, therefore, has implications for future researchers collecting information on children’s dietary intakes. To improve accuracy, researchers should adopt strategies to train children to provide better recounts of their dietary intakes. These include reminding children to pay attention to details of their meals, particularly side dishes, or encouraging children to complete the recall of their school meal after school instead of at the end of the day to facilitate memory retention. Future research should also investigate the development and applications of memory aids that may facilitate children in their dietary recalls, especially for less salient food items such as side dishes or accompaniments to their main meals and portion size estimations.

The present study has several strengths. The detailed analysis of reporting accuracy provides valuable insight into the validity of self-reported dietary information among children in the Asian context using a web-based application. The use of meal photography provides an objective reference standard to assess the accuracy of reported dietary intakes [25]. In principle, meal photography is similar to in-person meal observation, the most commonly used reference method for assessing the accuracy of self-reports, except that food intake is photographed by researchers before and after consumption, instead of being observed and recorded by trained observers in real-time [25]. Participants recruited from both study sites were of the same education level and similar age, reducing biases related to developmental and cognitive differences among children of different ages [31]. A consistent data collection methodology was also ensured through the development and use of a standard operating procedure. This minimised potential confounders, which may have explained the statistically insignificant differences in reporting accuracy between study sites.

This study is not without limitations. The sample size of the present study was small, so power was limited. We also note that participants were recruited from two schools and thus, findings may not be generalisable to other populations. The validation study only assessed school recess meals, limiting the ability to extrapolate the accuracy of the children’s self-reports for meals consumed outside school [11]. However, as the school environment provided a logistically feasible and least intrusive condition for assessing dietary intake objectively [27], it was the most apparent choice of location for the validation study. The response rate for the present study was notably low (59%), mainly contributed by the exclusion of participants who did not report their recess meal and/or complete entries on MEDAL. Tests for differences between excluded and included participants however identified no differences between them, suggesting that despite low response rates, the included participants remain representative of the recruited cohort.

## 5. Conclusions

Overall, children in Singapore aged between 10 to 11-years-old are able to report their school recess meals using MEDAL, although with varying degrees of accuracy. Absolute information on dietary intakes (food item and quantity) collected using MEDAL are to be interpreted with caution. Findings of the present study suggest that necessitating strategies to train and instruct the children to report their dietary intake in detail, particularly side dishes of their main meals, may improve the quality of self-reported dietary data collected. We propose further validation studies in larger samples that take into consideration meals consumed outside school, which will provide more insight to improving the validity of self-reported dietary intakes using MEDAL.

## Figures and Tables

**Table 1:** Demographic characteristics of Primary 5 students participating in the MEDAL diet validation study (*n* = 33).

	Total (*n* = 33)	School A (*n* = 24)	School B (*n* = 9)	*p*-Value ^1^
Age, years (interquartile range)	11.0 (11.0–11.0)	10.0 (10.0–11.0)	11.0 (11.0–11.0)	0.103
Sex, %				0.943
*Females*	54.5	54.2	55.6	
*Males*	45.5	45.8	44.4	
BMI-for-age %				0.720
*Underweight*	6.1	4.2	11.1	
*Healthy weight*	78.8	79.2	77.8	
*Overweight*	15.1	16.6	11.1	
Internet access, %				0.108
*Yes*	87.9	91.7	77.8	
*No*	12.1	8.3	22.2	
Days of recording, %				0.160
*1 day*	63.6	70.8	44.4	
*2 days*	36.4	29.2	55.6	

^1^ Difference in distribution of characteristics was assessed using Pearson χ^2^ tests.

**Table 2:** Reporting accuracy for food items by comparing recess meals reported in MEDAL with recess meal photos ^1^.

		Food Item Match						Omission ^4^		Intrusion ^5^	
		Strict ^2^		Loose ^3^		Total					
	Total *n*	*n*	%	*n*	%	*n*	%	*n*	%	*n*	%
Total	118	58	49.2	13	11.0	71	60.2	29	24.6	18	15.2
By food group											
*Rice and porridge*	11	5	45.5	4	36.4	9	81.8	2	18.2	0	0.0
*Breads, spreads and cereals*	18	13	72.2	1	5.6	14	77.8	0	0.0	4	22.2
*Noodles, pasta and potatoes*	13	9	69.2	1	7.7	10	76.9	1	7.7	2	15.4
*Drinks*	18	9	50.0	2	11.1	11	61.1	1	5.6	6	33.3
*Meat and fish*	20	9	45.0	3	15.0	12	60.0	7	35.0	1	5.0
*Fruits*	4	2	50.0	0	0.0	2	50.0	2	50.0	0	0.0
*Curry*	8	4	50.0	0	0.0	4	50.0	4	50.0	0	0.0
*Vegetables and mushrooms*	11	4	36.4	1	9.1	5	45.5	6	54.5	0	0.0
*Snacks and desserts*	7	2	28.6	1	14.3	3	42.9	1	14.3	3	42.9
*Eggs and tofu*	7	1	14.3	0	0.0	1	14.3	4	57.1	2	28.6
*Milk, cheese and yogurt*	1	0	0.0	0	0.0	0	0.0	1	100.0	0	0.0

^1^ Categories arranged in descending order of total match rates. ^2^ Strict matches occurred when a food item reported in MEDAL exactly matched the item in the meal photo. ^3^ Loose matches occurred when a food item reported in MEDAL was not identical to the item in the meal photo but belonged to the same food group. ^4^ Omission occurred when a food item was not reported in MEDAL but was present in the meal photo. ^5^ Intrusion occurred when a food item was reported in MEDAL but was not present in the meal photo.

**Table 3:** Reporting accuracy for portion sizes by comparing recess meals reported in MEDAL with recess meal photos ^1^.

		Portion Size Match		Underestimation		Overestimation	
	Total *n*	*n*	%	*n*	%	*n*	%
Total	48	28	58.3	4	8.3	16	33.3
By food group							
*Eggs and tofu*	1	1	100.0	0	0.0	0	0.0
*Drinks*	10	9	90.0	1	10.0	0	0.0
*Meat and fish*	9	7	77.8	0	0.0	2	22.2
*Vegetables and mushrooms*	3	2	66.7	0	0.0	1	33.3
*Breads, spreads and cereals*	8	5	62.5	1	12.5	2	25.0
*Fruits*	2	1	50.0	1	50.0	0	0.0
*Snacks and desserts*	2	1	50.0	1	50.0	0	0.0
*Rice and porridge*	5	1	20.0	0	0.0	4	80.0
*Noodles, pasta and potatoes*	7	1	14.3	0	0.0	6	85.7
*Curry*	1	0	0.0	0	0.0	1	100.0
*Milk, cheese and yogurt*	0	0	-	0	-	0	-

^1^ Categories arranged in descending order of portion matches.

## Data Availability

Data may be made available upon request to the corresponding author.

## Appendix A

**Table A1:** Inter-rater agreement on food item and portion sizes using kappa and weighted kappa respectively ^1^.

Food Group	Food Item		Portion Size	
	*n*	κ ^2^	*n*	κ ^2^
Overall	92	0.85	81	0.70
*Drinks*	16	0.55	13	NA ^3^
*Meat and fish*	28	0.90	27	0.62
*Noodles, pasta and potatoes*	12	0.88	11	0.62
*Rice and porridge*	9	0.42	9	NA ^3^
*All others* ^4^	27	0.83	21	0.90

^1^ Students of 9–10-years-old (*n* = 11) and 11–12-years-old (*n* = 10) participated in the pilot diet validation study, where they reported their recess meals on MEDAL and had their recess meals photographed on two weekdays. Two researchers analysed the recess meal photos and selected the food item and portion size option in MEDAL that was closest to what the child consumed. ^2^ Kappa (κ) of 0.10–0.20 indicated slight agreement, 0.21–0.40 indicated fair agreement, 0.41–0.60 indicated moderate agreement, 0.61–0.80 indicated substantial agreement, 0.81–0.99 indicated near-perfect agreement, and 1.00 indicated perfect agreement. ^3^ No statistics are computed because the portion size selected by either one or both researchers is/are constant(s). ^4^ Food groups with less than nine occurrences among all participants were collapsed onto an “All others” category. These food groups are: Breads, spreads and cereals; Eggs and tofu; Fast Food; Fruits; Snacks and desserts; and Vegetables and mushrooms.

**Table A2:** Demographic characteristics of Primary 5 students included and excluded from the MEDAL diet validation study (*n* = 56).

	Included (*n* = 33)	Excluded (*n* = 23)	*p*-Value ^1^
Age, years (interquartile range)	11.0 (10.0–11.0)	11.0 (10.0–11.0)	0.586
School, %			
*School A*	72.7	52.2	0.114
*School B*	27.3	47.8	
Sex, %			
*Females*	54.5	47.8	0.621
*Males*	45.5	52.2	
BMI-for-age, %			
*Underweight*	26.1	4.3	0.377
*Healthy range*	78.8	87.0	
*Overweight*	15.2	8.7	
Internet access, %			
*Yes*	90.9	82.6	0.355
*No*	9.1	17.4	

^1^ Difference in distribution of characteristics was assessed using Pearson χ^2^ tests.

**Table A3:** Reporting accuracy for food items by comparing recess meal photos with recess meals reported in MEDAL, stratified by sex, school, and day of recording, presented as median (interquartile range) ^1^.

	Food Item Match			Omission ^4^ (%)	Intrusion ^5^ (%)
	Strict ^2^ (%)	Loose ^3^ (%)	Total (%)		
By sex					
*Males (n = 15)*	50.0 (0.0–80.0)	0.0 (0.0–0.0)	60.0 (25.0–100.0)	0.0 (0.0–40.0)	0.0 (0.0–40.0)
*Females (n = 18)*	50.0 (33.3–75.0)	0.0 (0.0–25.0)	75.0 (44.4–100.0)	0.0 (0.0–50.0)	0.0 (0.0–0.0)
*p-value* ^1^	*0.985*	*0.112*	*0.435*	*0.692*	*0.230*
By school					
*School A (n = 24)*	50.0 (23.6–55.0)	0.0 (0.0–21.1)	55.0 (38.9–100.0)	0.0 (0.0–50.0)	0.0 (0.0–25.0)
*School B (n = 9)*	66.7 (50.0–80.0)	0.0 (0.0–0.0)	80.0 (60.0–100.0)	20.0 (0.0–33.3)	0.0 (0.0–0.0)
*p-value* ^1^	*0.175*	*0.680*	*0.361*	*0.965*	*0.551*
By day of recording					
*First day (n = 12)*	50.0 (26.7–100.0)	0.0 (0.0–0.0)	75.0 (33.3–100.0)	0.0 (0.0–50.0)	0.0 (0.0–0.0)
*Second day (n = 12)*	70.8 (29.2–100.0)	0.0 (0.0–25.0)	87.5 (58.3–100.0)	0.0 (0.0–29.2)	0.0 (0.0–0.0)
*p-value* ^1^	*0.367*	*0.564*	*0.469*	*0.352*	*0.951*

^1^ Differences in food item reporting accuracy were assessed by Mann–Whitney test for sex and school variables, and Wilcoxon matched-pairs signed-rank test for day of recording variable. ^2^ Strict matches occurred when a food item reported in MEDAL exactly matched the item in the meal photo. ^3^ Loose matches occurred when a food item reported in MEDAL was not identical to the item in the meal photo but belonged to the same food group. ^4^ Omission occurred when a food item was not reported in MEDAL but was present in the meal photo. ^5^ Intrusion occurred when a food item was reported in MEDAL but was not present in the meal photo.

## References

[1] Kelder S.H., Perry C.L., Klepp K.I., Lytle L.L. (1994). Longitudinal tracking of adolescent smoking, physical activity, and food choice behaviors. Am. J. Public Health.

[2] Baxter S.D., Hitchcock D.B., Royer J.A., Smith A.F., Guinn C.H. (2017). Fourth-grade children’s dietary reporting accuracy by meal component: Results from a validation study that manipulated retention interval and prompts. Appetite.

[3] Lu A.S., Baranowski J., Islam N., Baranowski T. (2014). How to engage children in self-administered dietary assessment programmes. J. Hum. Nutr. Diet..

[4] de Vlieger N.M., Weltert M., Molenaar A., McCaffrey T.A., Rollo M.E., Truby H., Livingstone B., Kirkpatrick S.I., Boushey C.J., Kerr D.A. (2020). A systematic review of recall errors associated with portion size estimation aids in children. Appetite.

[5] Illner A.K., Freisling H., Boeing H., Huybrechts I., Crispim S.P., Slimani N. (2012). Review and evaluation of innovative technologies for measuring diet in nutritional epidemiology. Int. J. Epidemiol..

[6] Baranowski T., Islam N., Baranowski J., Cullen K.W., Myres D., Marsh T. (2002). The food intake recording software system is valid among fourth-grade children. J. Am. Diet. Assoc..

[7] Moore H.J., Ells L.J., McLure S.A., Crooks S., Cumbor D., Summerbell C.D., Batterham A.M. (2008). The development and evaluation of a novel computer program to assess previous-day dietary and physical activity behaviours in school children: The Synchronised Nutrition and Activity Program (SNAP). Br. J. Nutr..

[8] Storey K.E., McCargar L.J. (2012). Reliability and validity of Web-SPAN, a web-based method for assessing weight status, diet and physical activity in youth. J. Hum. Nutr. Diet..

[9] Biltoft-Jensen A., Bysted A., Trolle E., Christensen T., Knuthsen P., Damsgaard C.T., Andersen L.F., Brockhoff P., Tetens I. (2013). Evaluation of web-based dietary assessment software for children: Comparing reported fruit, juice and vegetable intakes with plasma carotenoid concentration and school lunch observations. Br. J. Nutr..

[10] Diep C.S., Hingle M., Chen T.A., Dadabhoy H.R., Beltran A., Baranowski J., Subar A.F., Baranowski T. (2015). The automated self-administered 24-hour dietary recall for children, 2012 version, for youth aged 9 to 11 years: A validation study. J. Acad. Nutr. Diet..

[11] Davies V.F., Kupek E., de Assis M.A., Natal S., Di Pietro P.F., Baranowski T. (2015). Validation of a web-based questionnaire to assess the dietary intake of Brazilian children aged 7–10 years. J. Hum. Nutr. Diet..

[12] Sugianto R., Chan M.J., Wong S.F., Shek L.P.C., Tan K.H., Chong Y.S., Godfrey K.M., Tai B.C., Chong M.F.F. (2020). Evaluation of a quantitative food frequency questionnaire for 5-year-old children in an asian population. J. Acad. Nutr. Diet..

[13] Health Promotion Board Students’ Health Survey. https://data.gov.sg/dataset/students-health-survey.

[14] Health Promotion Board National Nutrition Survey 2010. https://www.hpb.gov.sg/docs/default-source/pdf/nns-2010-report.pdf?sfvrsn=18e3f172_2.

[15] Brownlee I.A., Low J., Duriraju N., Chun M., Ong J.X.Y., Tay M.E., Hendrie G.A., Santos-Merx L. (2019). Evaluation of the proximity of singaporean children’s dietary habits to food-based dietary guidelines. Nutrients.

[16] Chia A., Chew M.N.J.S., Tan S.Y.X., Chan M.J., Colega M.T., Toh J.Y., Natarajan P., Lança C., Shek L.P., Saw S.M. (2021). A web-based time-use application to assess diet and movement behavior in asian schoolchildren: Development and usability study of my e-diary for activities and lifestyle (MEDAL). J. Med. Internet Res..

[17] Poslusna K., Ruprich J., de Vries J.H.M., Jakubikova M., van’t Veer P. (2009). Misreporting of energy and micronutrient intake estimated by food records and 24 hour recalls, control and adjustment methods in practice. Br. J. Nutr..

[18] Baxter S.D., Smith A.F., Litaker M.S., Baglio M.L., Guinn C.H., Shaffer N.M. (2004). Children’s social desirability and dietary reports. J. Nutr. Educ. Behav..

[19] Baranowski T., Domel S.B. (1994). A cognitive model of children’s reporting of food intake. Am. J. Clin. Nutr..

[20] Foster E., Bradley J. (2018). Methodological considerations and future insights for 24-hour dietary recall assessment in children. Nutr. Res..

[21] Lillegaard I.T., Overby N.C., Andersen L.F. (2005). Can children and adolescents use photographs of food to estimate portion sizes?. Eur. J. Clin. Nutr..

[22] Krehbiel C.F., DuPaul G.J., Hoffman J.A. (2017). A Validation study of the automated self-administered 24-hour dietary recall for children, 2014 version, at school lunch. J. Acad. Nutr. Diet..

[23] Graziose M.M., Wolf R.L., Koch P.A., Gray H.L., Contento I.R. (2018). Validation of a questionnaire to measure fruits and vegetables selected and consumed at school lunch among second- and third-grade students. J. Acad. Nutr. Diet..

[24] Biltoft-Jensen A., Damsgaard C.T., Andersen R., Ygil K.H., Andersen E.W., Ege M., Christensen T., Sørensen L.B., Stark K.D., Tetens I. (2015). Accuracy of self-reported intake of signature foods in a school meal intervention study: Comparison between control and intervention period. Br. J. Nutr..

[25] Tugault-Lafleur C.N., Black J.L., Barr S.I. (2017). A Systematic review of methods to assess children’s diets in the school context. Adv. Nutr..

[26] Swanson M. (2008). Digital photography as a tool to measure school cafeteria consumption. J. Sch. Health.

[27] Lyng N., Fagt S., Davidsen M., Hoppe C., Holstein B., Tetens I. (2013). Reporting accuracy of packed lunch consumption among Danish 11-year-olds differ by gender. Food Nutr. Res..

[28] Sabinsky M.S., Toft U., Andersen K.K., Tetens I. (2013). Validation of a digital photographic method for assessment of dietary quality of school lunch sandwiches brought from home. Food Nutr. Res..

[29] Winzer E., Luger M., Schindler K. (2018). Using digital photography in a clinical setting: A valid, accurate, and applicable method to assess food intake. Eur. J. Clin. Nutr..

[30] Health Promotion Board Body Mass Index (BMI)-for-Age-Children and Youth Aged 6 to 18 Years Old. https://bukitbatoksec.moe.edu.sg/qql/slot/u537/Total%20Curriculum/Curricular%20Areas/PE/BMI_chart.pdf.

[31] Pérez-Rodrigo C., Artiach Escauriaza B., Artiach Escauriaza J., Polanco Allúe I. (2015). Dietary assessment in children and adolescents: Issues and recommendations. Nutr. Hosp..

[32] Raffoul A., Hobin E.P., Sacco J.E., Lee K.M., Haines J., Robson P.J., Dodd K.W., Kirkpatrick S.I. (2019). School-age children can recall some foods and beverages consumed the prior day using the automated self-administered 24-hour dietary assessment tool (ASA24) without assistance. J. Nutr..

[33] Willett W. (1998). Nutritional Epidemiology.

[34] Baxter S.D., Hitchcock D.B., Guinn C.H., Vaadi K.K., Puryear M.P., Royer J.A., McIver K.L., Dowda M., Pate R.R., Wilson D.K. (2014). A validation study concerning the effects of interview content, retention interval, and grade on children’s recall accuracy for dietary intake and/or physical activity. J. Acad. Nutr. Diet..

[35] Baxter S.D., Royer J.A., Guinn C.H., Hardin J.W., Smith A.F. (2009). Origins of intrusions in children’s dietary recalls: Data from a validation study concerning retention interval and information from school food-service production records. Public Health Nutr..

[36] Smith A.F., Baxter S.D., Hardin J.W., Royer J.A., Guinn C.H. (2008). Some intrusions in dietary reports by fourth-grade children are based on specific memories: Data from a validation study of the effect of interview modality. Nutr. Res..

[37] Guinn C.H., Baxter S.D., Hardin J.W., Royer J.A., Smith A.F. (2008). Intrusions in children’s dietary recalls: The roles of BMI, sex, race, interview protocol, and social desirability. Obesity.

[38] Champagne C.M., Baker N.B., DeLany J.P., Harsha D.W., Bray G.A. (1998). Assessment of energy intake underreporting by doubly labeled water and observations on reported nutrient intakes in children. J. Am. Diet. Assoc..

[39] Smith A.F., Baxter S.D., Hitchcock D.B., Finney C.J., Royer J.A., Guinn C.H. (2016). Cognitive ability, social desirability, body mass index and socioeconomic status as correlates of fourth-grade children’s dietary-reporting accuracy. Eur. J. Clin. Nutr..

[40] Klesges L.M., Baranowski T., Beech B., Cullen K., Murray D.M., Rochon J., Pratt C. (2004). Social desirability bias in self-reported dietary, physical activity and weight concerns measures in 8-to 10-year-old African-American girls: Results from the girls health enrichment multisite studies (GEMS). Prev. Med..

[41] Carvalho M.A., Baranowski T., Foster E., Santos O., Cardoso B., Rito A., Pereira Miguel J. (2015). Validation of the Portuguese self-administered computerised 24-hour dietary recall among second-, third- and fourth-grade children. J. Hum. Nutr. Diet..

